# Luminal Sweet Sensing and Enteric Nervous System Participate in Regulation of Intestinal Glucose Transporter, GLUT2

**DOI:** 10.3390/nu17091547

**Published:** 2025-04-30

**Authors:** Andrew W. Moran, Miran Alrammahi, Kristian Daly, Darren Weatherburn, Catherine Ionescu, Alexandra Blanchard, Soraya P. Shirazi-Beechey

**Affiliations:** 1 Institute of Infection, Veterinary and Ecological Sciences, University of Liverpool, Liverpool L69 7ZJ, UK; andrew.moran@liverpool.ac.uk (A.W.M.); nkd@liverpool.ac.uk (K.D.); weatherburn.d@googlemail.com (D.W.); 2 Department of Physiology, Biochemistry and Pharmacology, College of Veterinary Medicine, University of Al-Qadisiyah, Al-Diwaniyah 58002, Iraq; 3 ADM International Sarl, 1180 Rolle, Switzerland; catherine.ionescu@adm.com (C.I.); alexandra.blanchard@adm.com (A.B.)

**Keywords:** luminal nutrient sensing, T1R2–T1R3, sweet taste, enteric nervous system, GLUT2, regulation, intestine, dietary additives, plant-based sweeteners, nutrition

## Abstract

**Background/Objectives:** Dietary glucose is transported across the intestinal absorptive cell into the systemic circulation by the apically located Na^+^-dependent glucose transporter 1 (SGLT1, *SLC5A1*) and basally residing Na^+^-independent glucose transporter 2 (GLUT2, *SLC2A2*). Whilst recent experimental evidence has shown that sensing of sweet compounds by the gut-expressed sweet taste receptor T1R2–T1R3 and glucagon-like peptide-2 receptor signalling are components of the pathway controlling SGLT1 expression, little is known about the mechanisms involved in the regulation of GLUT2. In this study, we tested the hypothesis that T1R2–T1R3 and its downstream signalling pathway participate in the regulation of intestinal GLUT2. **Methods**: We used in vivo and in vitro approaches employing a weaning pig model, a heterologous expression assay, and knockout mice for elucidating the regulation of GLUT2 by luminal sugars. **Results**: A plant-based sweetener formulation included in piglets’ diet led to a marked increase in GLUT2 expression in piglets’ intestine, compared to controls. The sweeteners that do not activate pig T1R2–T1R3 failed to upregulate GLUT2. There was a significant increase in GLUT2 expression when the sweetener sucralose, which activates T1R2–T1R3, was included in the drinking water of wild-type mice. However, in knockout mice, in which the genes for the sweet receptor subunit T1R3 and the associated G-protein gustducin were deleted, there was no upregulation of GLUT2 expression in response to sucralose supplementation. There was a notable increase in GLUT2 expression in wild-type mice fed a high-carbohydrate diet compared to when maintained on a low-carbohydrate diet. However, in GLP-2 receptor knockout mice kept on the high-carbohydrate diet, there was no enhancement in GLUT2 expression. **Conclusions**: The experimental evidence suggests that luminal sweet sensing via T1R2–T1R3 and the enteroendocrine-derived GLP-2 are constituents of the regulatory pathway controlling GLUT2 expression.

## 1. Introduction

The accepted model of glucose absorption across the intestinal epithelium is that glucose absorbed by the apically located SGLT1 exits the cell across the basolateral membrane into the systemic circulation by glucose transporter GLUT2 [1,2,3,4]. Absorption of glucose is highest in the proximal intestine (duodenum and jejunum). Using a glucose concentration gradient, GLUT2 can transport glucose out of or into the cell [5]. In patients with Fanconi Bickle syndrome with a mutation in *GLUT2* gene, there is an impairment in glucose transport [5,6], supporting the participation of GLUT2 in intestinal glucose transport. Understanding the regulatory pathway controlling transcellular transport of glucose in the intestine via SGLT1 and GLUT2 is important for the provision of optimal energy and maintenance of glucose homeostasis.

The gut epithelium can sense sugars and low-calorie (non-nutritive, artificial) sweeteners via the gut-expressed G-protein coupled sweet taste receptor, Taste 1 Receptor 2–Receptor 3 (T1R2–T1R3), expressed in L-endocrine cells to modulate Na^+^-dependent glucose absorptive capacity [7,8]. Furthermore, it has been shown that T1R2 and T1R3, together with the associated G-protein, α-gustducin, are co-expressed in L-endocrine cells containing GLP-1 and GLP-2 [7]. L-cells secrete GLP-1 and GLP-2 in response to luminal glucose and sweeteners [7,9,10], and have been identified throughout the intestinal tract, with the highest numbers observed in the distal intestine [11]. However, the proximal intestine has the greatest density of GLP-2 receptor cells [12] and shows the largest response to pharmacological stimulation of the GLP-2 receptor [13,14]. GLP-2 regulates intestinal growth, nutrient (e.g., glucose, amino acid) absorption, and barrier function [15,16,17,18]. Intravenous or serosal application of GLP-2 results in increased SGLT1 expression, maximal rate of Na^+^-dependent glucose transport, and a corresponding increase in blood glucose concentration [17,19,20,21].

We have described the pathway by which sensing of dietary monosaccharides/artificial sweeteners regulates SGLT1 expression and activity, by demonstrating that sensing of sweet compounds by the gut-expressed sweet taste receptor T1R2–T1R3 evokes secretion of GLP-2 from enteroendocrine L-cells. This gut hormone, when bound to its receptor on the enteric neurons, elicits the release of neuropeptide VIP or PACAP [10]. The binding of the neuropeptide to its receptor, a stimulatory G-protein VPAC1, enhances intracellular cAMP and the half-life of SGLT1 mRNA [10,22,23] resulting in an increase in SGLT1 expression and activity [10]. Little convincing evidence is available on the regulatory pathway controlling the expression of the intestinal basolateral membrane glucose transporter, GLUT2.

In this paper, we have aimed to identify constituents of the pathway regulating GLUT2 expression. We have used in vivo and in vitro approaches employing a weaning pig model, a heterologous expression assay, and knockout mice for elucidating the regulation of GLUT2 by luminal sugars. The data propose that T1R2–T1R3 and GLP-2 participate in the regulation of the intestinal basolateral membrane glucose transporter, GLUT2.

## 2. Materials and Methods

### 2.1. Animals

#### 2.1.1. Piglets, Dietary Trials, Tissue Collection

To assess the effect of a diet containing a plant-based sweetener formulation (brand name SMF, ADM, Rolle, Switzerland) on the expression and activity of GLUT2 (and SGLT1), sixteen 28-day-old male and female suckling (Gloucester Old Spot) piglets were used in this study. They were maintained in pens measuring 1.5 × 1.5 m^2^ with 2 piglets per pen. The pens were kept in a room maintained at 26.7 °C and exposed to a 12 h light and dark cycle. Dust-extracted wood shavings were used as bedding to avoid unwanted dietary contributions. The piglets were weaned onto either a basal diet (composition of the diet is described in Table S1) or the basal diet containing sweetener formulation SMF. SMF contains vanillin, neohesperidin dihydrochalcone (NHDC), glycyrrhizinate, Stevia extract, monkfruit and fenugreek extract. These natural plant extracts are used as ingredients in the human diet, or some as sweeteners for human usage. Piglets were maintained on these diets for 14 days and observed throughout the study. They remained healthy without any visible enteric disorders.

#### 2.1.2. Effect of Sweeteners, with Differential Effect on Activating Pig T1R2–T1R3, on Activity and Expression of GLUT2

To determine the effect of sweeteners sucralose, cyclamate and aspartame on SGLT1 and GLUT2 activity and expression, 4 groups of 28-day-old male and female suckling piglets (Landrace X Large White, each group consisting of 4 piglets) were maintained on the basal diet (for composition, see Table S1) for 3 days, with either sucralose (2 mM), cyclamate (10 mM) or aspartame (1 mM) added to their drinking water. The fourth group consumed drinking water alone. The concentration of sweeteners used was based on a preference test as described in Glaser et al., 2000 [24].

#### 2.1.3. Tissue Collection and Usage

At the end of experiments described above piglets were euthanised using an intravenous injection of 20 mL of pentobarbitone (200 mg/mL Dolethal; Vetoquinol UK Limited, Northants, UK) into the cranial vena cava (in line with the UK Home Office Schedule 1 regulation) by a qualified veterinarian.

Immediately after euthanasia, approximately 40 cm length sections of the proximal intestine were removed, everted and rinsed in ice-cold saline. Mucosal scrapings were collected from segments of fresh everted proximal intestinal tissue by gently scraping the mucosa using a glass slide. Sections of everted tissue and mucosal scrapings were immediately frozen in liquid nitrogen. They were stored at −80 °C until used for isolation of brush border and basolateral membrane vesicles used for glucose uptake studies, and western blot analysis outlined in this study.

Two-centimetre sections of proximal small intestine from the same regions described above were fixed in 10% neutral buffered formalin for 4 h, and then cryoprotected in 20% (*w*/*v*) sucrose (Fluka, Gillingham, Dorset, UK) in PBS overnight before embedding in OCT and storing at −80 °C. They were used for immunohistochemical studies and morphometric analysis.

#### 2.1.4. Large-Scale Feeding Trial

In a large-scale feeding trial, eighty 22-day-old male and female piglets (40 males:40 females; PIC 1050 Camborough × 337 line cross) were weaned to three-phase diets consecutively for 28 days. Forty piglets were weaned to diets D1, D2 and D3 (control group) and 40 were given diets D1, D2 and D3 + 100 ppm SMF (experimental group) (see Table S2 for the composition of diets, D1, D2 and D3). Diets were isocaloric. Piglets were housed in pens (1.62 m^2^) with four piglets per pen, in two rooms maintained initially at 30 °C and gradually reducing the temperature to 26 °C. Their health and performance were monitored regularly.

#### 2.1.5. Mice, Diets and Tissue Collection

Intestinal tissue samples used in this study were from C57BL/6 G_α_-gustducin and T1R3^−/−^ mice generated for our study described in detail in Margolskee et al., 2007 [7]. Briefly, six- to eight-week-old C57BL/6 G_α_-gust^−/−^ or T1R3^−/−^ male and female mice (similar numbers of each sex) and their wild-type littermates of the same background (8 in each group) were placed individually in cages as described before [7]. Group one was fed a high-carbohydrate diet (69.8% sucrose, energy 3.77 kcal/g Test diet #5810, Purina Mills, St. Louis, MO, USA), group two were fed a low-carbohydrate diet (1.9% sucrose) having similar energy 3.86 kcal/g Test diet #590N, Purina Mills, and group three were maintained on the low-carbohydrate diet with their drinking water supplemented with sucralose (2 mM) for 14 days.

The creation of the C57BL/6 GLP-2 receptor knockout (Glp2r^−/−^) mice and experimental details have been reported before [10,25]. Tissues used in this study were shared with our previous study [10]. C57BL/6 Glp2r^−/−^ male and female mice (similar numbers of each sex) and their wild-type littermates of the same background (8 in each group) were placed individually in cages as described before [10]. Group one was fed the high-carbohydrate diet (Test diet #5810) and group two was maintained on the low-carbohydrate diet (Test diet #590N) for 5 days.

All mice were killed by cervical dislocation in accordance with the UK Animals (Scientific Procedures) Act, 1986 and with guidelines set out by the University of Liverpool Ethics Committee and in line with UK Home Office Schedule 1 regulations. Immediately after death the proximal small intestine was removed, everted and rinsed in ice-cold saline to remove the contents. Sections of the proximal (mid) intestine were either used fresh for electric field stimulation experiments or were frozen immediately in liquid nitrogen for RNA analysis.

### 2.2. Taste Receptor Constructs

Full-length coding sequences of pig *Tas1R2* and *Tas1R3* genes were custom synthesised and ligated into modified pcDNA3/TO vector with the addition of amino-terminal sst-tags (rat somatostatin receptor subtype 3) to facilitate cell membrane targeting and carboxy-terminal FLAG or HSV tags to enable immunological detection of each receptor subunit as described before [26].

### 2.3. Immunocytochemistry

For determining T1R2 and T1R3 expression in transfected cells, immunocytochemistry was conducted as outlined previously [26]. Human embryonic kidney HEK293PEAKrapid cells that stably express G_α15_ were transiently transfected with pig sweet receptor constructs or with an empty vector (to act as negative control) as detailed previously [26]. T1R2 and T1R3 proteins were detected using anti-FLAG (anti-DDDDK tag antibody rabbit polyclonal, ab-1162, Abcam, Cambridge, UK) and anti-HSV (goat polyclonal, ab-19345, Abcam, UK) primary antibodies 1:15,000 dilution. Cells were further incubated with fluorescein (FITC)-conjugated donkey anti-rabbit or Cy3-conjugated donkey anti-goat IgG, and subsequently mounted and visualised. Images were captured with a DS-Fi3 fluorescent camera (Nikon Instruments, Surrey, UK) with merging images performed using NIS-Elements Basic Research software (vs 5.30.04, Nikon Instruments, UK).

### 2.4. Heterologous Expression Assay

To evaluate pig T1R2–T1R3 sweetener activation, we used our previous methodology [26]. Briefly, HEK293PEAKrapid G_α_15 cells were seeded into poly-D-lysine coated 96-well plates, transfected with either pig sweet receptor constructs or empty vector, and were loaded with Ca^2+^ sensitive Fluo-4AM fluorescent dye. Forty-eight compounds were assessed for their abilities to stimulate pig T1R2–T1R3. The selected sweeteners consisted of vanillin, NHDC, glycyrrhizinate, stevia extract, monk fruit and fenugreek extract (assessed first singly and subsequently in combination). Calcium responses of transfected cells upon sweetener application were measured using FlexStation3 microplate reader (Molecular Devices UK Ltd., Wokingham, UK). Plots of signal amplitude (ΔF/F) against concentration were used to determine the EC_50_ values, as described before [26]. The EC_50_ values were calculated by nonlinear regression of amplitude plots to the function f(x) = 100/[1 + (EC_50_/x)^nH^], where x is the agonist concentration and nH is the Hill coefficient [27].

### 2.5. Electric Field Stimulation (EFS)

This procedure is based on a previously described publication [28], with some changes in the methodology for electrically stimulating enteric neurons [10]. Fresh whole mouse proximal intestinal tissues maintained in oxygenated (Carbogen, BOC, Worsley, UK) Krebs-Henseleit buffer at 37 °C were flushed with the buffer and divided into 3 cm loops. The tissue loops kept vertical as described before [10] were maintained in a tissue bath containing Krebs-Henseleit buffer fitted with a water jacket maintained at 37 °C. They were stimulated electrically with one electrode placed inside the intestinal loop at ~3 mm to the luminal membrane and the other outside the loop at the same distance. Electrical current was applied using square wave pulses [10]. The unstimulated tissue loops served as controls. One set of tissue loops that were stimulated electrically was preincubated with 10 µM tetrodotoxin (TTX) before stimulation in TTX-containing buffer.

After the electric stimulation, the tissue was wrapped in aluminium foil and frozen immediately in liquid nitrogen and stored at −80 °C for mRNA isolation. In all three sets of tissues, control and stimulated ± TTX, tissue integrity and profile of GLUT2 expression on the basolateral membrane of villus enterocytes were confirmed by immunohistochemistry.

### 2.6. Isolation of Brush Border Membrane Vesicles

All steps in the preparation of brush border membrane vesicles (BBMV) from pig everted and frozen intestinal tissues were performed on ice or at 4 °C according to procedures described by Shirazi-Beechey et al., 1990 [29] with modifications outlined by Moran et al., 2018 [10]. Magnesium precipitation was used to aggregate basolateral, organelle and nuclear membrane followed by centrifugation at 3000× *g* to pellet the aggregated membranes. The supernatant containing BBMV was spun twice at 30,000× *g* and the final pellet containing purified BBMV was homogenised in an isotonic buffer. The protein concentration of BBMV was determined using Bio-Rad assay [29]. Aliquots of freshly prepared BBMV were diluted with sample buffer (62.5 mM Tris/HCl pH 6.8, 10% (*v*/*v*) glycerol, 2% (*w*/*v*) SDS, 0.05% (*v*/*v*) β-mercaptoethanol (M-7154, Sigma, St. Louis, MO, USA), 0.05% (*w*/*v*) bromophenol blue (035730, BDH)) and stored at −20 °C to be used for western blotting.

### 2.7. Isolation of Basolateral Membrane Vesicles

The isolation of basolateral membrane vesicles (BLMV) from pig frozen proximal intestinal tissues is based on the procedure described by Dyer et al., (2002) [30]. All steps in the procedure were carried out at 4 °C. In brief, cation precipitation, differential centrifugation and sorbitol density gradient were used to prepare BLMV. Frozen mucosal scrapings were thawed in a buffer and homogenised. The homogenate was centrifuged at 500× *g*, and the resulting supernatant was further centrifuged at 20,000× *g*. The pellet was resuspended in a hypotonic buffer [30] to which MgCl_2_ was added, at final concentration of 8 mM and the sample was stirred for 20 min on ice. The suspension was centrifuged at 2000× *g* for 10 min and pellet was resuspended in a hypotonic buffer. The resuspended pellet was homogenised and centrifuged at 20,000× *g* for 20 min. The resultant pellet was homogenised in sorbitol buffer and the sample was overlaid onto a 35–55% (*w*/*v*) sorbitol density gradient and centrifuged at 100,000× *g* for 60 min. The discrete white band nearer to the top of the gradient containing BLMV [30] was homogenised in an isotonic buffer. Subsequently, the homogenate was centrifuged at 100,000× *g* for 30 min. The final pellet was resuspended in the isotonic buffer and stored in aliquots in liquid nitrogen until use for glucose uptake and measurements of GLUT2 protein abundance.

### 2.8. Western Blot Analysis

The abundance of SGLT1 and GLUT2 proteins was determined by western blotting using antibodies against custom synthesised C-termini regions of SGLT1 [7] and GLUT2 [30] proteins as described before [7]. Protein components of BBMV and BLMV were separated by SDS-polyacrylamide gel electrophoresis on 8% (*w*/*v*) polyacrylamide mini gels, containing 0.1% (*w*/*v)* SDS, and electro-transferred to PVDF membrane (1620264, Immun-Blot, Bio-Rad Laboratories Ltd., Hemel Hempstead, UK). For the determination of SGLT1 protein abundance, membranes were incubated for 1 h at room temperature in a buffer consisting of PBS containing 0.5% (*w*/*v*) non-fat dried milk (LP0031, Oxoid, Hampshire, UK), and 0.1% (*v*/*v*) Tween-20 (20605 Fisher, Hampton, NH, USA), before exposure to SGLT1 antibody diluted 1:1000. For immunodetection of GLUT2 protein the same procedure was used with the exception that membranes were blocked with 5% (*w*/*v*) milk in PBS-TM before incubating with GLUT-2 antibody (1:1000). Immuno-reactive bands were identified using affinity purified horseradish peroxidase-linked anti-rabbit secondary antibody diluted 1:2000 in PBS-TM. Protein bands were visualised with Immobilon Western Chemiluminescent HRP Substrate followed by scanning densitometry. β-actin was used as a loading control.

### 2.9. Glucose Transport into Membrane Vesicles

Uptake of 0.1 mM [U-^14^C]-D-glucose by BBMV and 1mM [U-^14^C]-D-glucose by BLMV was determined by rapid filtration stop technique, as reported before [10,30]. Initial rates of ^14^C glucose transport into BBMV, and BLMV were determined in the presence of either 100 mM sodium/potassium thiocyanate or mannitol, respectively.

### 2.10. Immunohistochemistry

Immunohistochemistry was performed as previously reported [10]. In brief, proximal intestinal tissue sections embedded in OCT were sectioned at a thickness of 5–7 μm onto poly-L-lysine coated slides. Subsequently, the slides were washed in PBS followed by incubation in blocking solution (10% (*v*/*v*) donkey serum in PBS) at 25 °C. Sections were then incubated overnight at 4 °C with primary antibodies against custom synthesised C-termini regions of SGLT1 and GLUT2 proteins, both diluted 1:100. After washing, the slides were exposed to FITC-conjugated donkey anti-rabbit secondary antibody diluted 1:500. Finally, the slides were mounted in Vectashield hard set mounting media with DAPI. The immunostaining was visualised using an epifluorescence microscope (Nikon, UK) and images were captured with a DS-Fi3 fluorescent camera (Nikon Instruments, UK) using NIS-Elements Basic Research software (vs 5.30.04, Nikon Instruments, UK). For sections used as controls primary antibodies for SGLT1 or GLUT2 were omitted.

### 2.11. Morphometric Analysis

Morphometric analysis was performed as described previously [10]. Sections, 10 µm thick were exposed to Mayer’s Haemalum and stained with eosin Y solution. After being dehydrated by stepwise washing in 70% ethanol (*v*/*v*), absolute ethanol and xylene they were mounted with D.P.X. neutral mounting medium. Digital images were captured with an Eclipse E400 microscope and DS-Fi3 camera (Nikon, UK), analysed using ImageJ software (vs 1.53v) and calibrated. The villus height and crypt depth measurements were taken from an average of sixteen well-oriented crypt-villus units. All images were captured under the same conditions with care taken to ensure that the same villus was not counted again.

### 2.12. Quantitative PCR

Expression of GLUT2 mRNA was assessed by quantitative PCR as described before [10]. RNA was isolated and used as template for first-strand cDNA synthesis as detailed previously [10]. cDNA was purified using QiaQuick PCR purification kit and qPCR assays were performed using 25 ng cDNA as template per 25 µL reaction containing SYBR Green JumpStart Taq ReadyMix for qPCR and 900 nM of each primer. RNA polymerase IIA (POLR2A), β-actin (ACTB), β2-microglobulin (B2M) were used as controls.

### 2.13. Statistics

D’Agostino and Pearson omnibus and Shapiro–Wilk normality tests were used to confirm that data were normally distributed. A Student’s two-tailed *t*-test was employed to determine statistical significance (GraphPad Prism 5, GraphPad Software Inc., La Jolla, CA, USA) for the following: comparing SGLT1 and GLUT2 expression, measurements of crypt depth/villus height in intestinal tissues, and differences in the growth parameters of piglets maintained on control diet and the diet containing SMF. For the large-scale animal performance trial, data were analysed using JMP Pro 17.0.0 with an ANOVA considering the effect of the treatment and piglet initial body weight block. A one-way ANOVA with Dunnett’s multiple comparison post-test (GraphPad Prism 5) was applied for data on expression of GLUT2 in the pig intestine after inclusion of artificial sweeteners in their drinking water and data arising from EFS experiments. The level of statistically significant was set at *p* < 0.05.

## 3. Results

### 3.1. Heterologous Expression of Pig T1R2–T1R3 and Response to Sweetener SMF

It is established, using in vivo and in vitro strategies that T1R2−T1R3 combination functions as the sweet receptor [31,32]. Moreover, the receptor resides on the apical membrane of enteroendocrine cells [27]. As such, before assessing if a sweetener can activate pig sweet receptors, using heterologous expression assay, it was necessary to show that T1R2, T1R3 are co-expressed and reside at the cell membrane (see Supplementary Figure S1). To demonstrate if sweetener formulation, SMF, activates pig T1R2–T1R3, we assessed the response of pig T1R2–T1R3 to SMF using HEK29PEAKrapid cells stably expressing the G-protein subunit G_α15_. The association of expressed taste receptor subunits with G_α15_ couples the extracellular activation of the functional sweet receptor to an elevation of cytosolic Ca^2+^ concentrations, allowing calcium fluorescence response to be measured [26]. The signal amplitude of Ca^2+^ fluorescence (ΔF/F) in the T1R2/T1R3 transfected cells was expressed relative to cells transfected with the empty vector.

**Figure 1:**
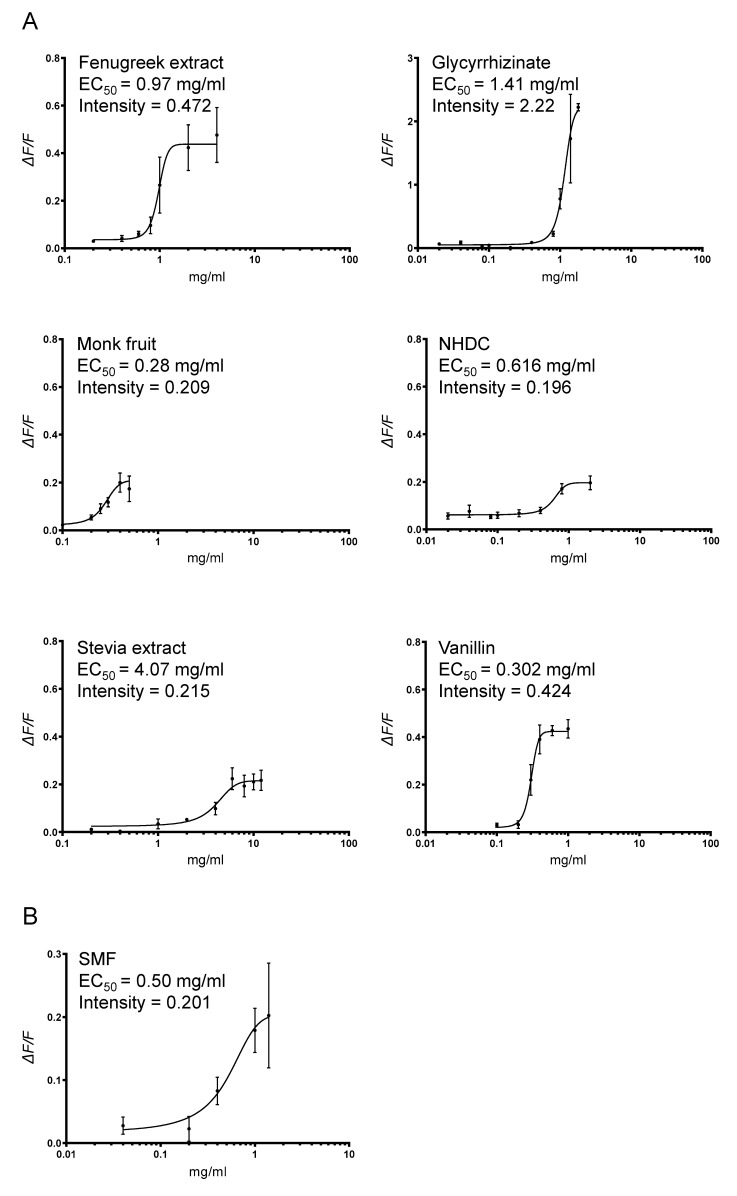
Activation of pig T1R2/T1R3 by SMF. Concentration–response curves of pig T1R2–T1R3 activation in response to stimulation with either each sweetener component of SMF (**A**) or the SMF formulation containing all the components (**B**) are shown. Signal amplitude of Ca^2+^ fluorescence (ΔF/F) versus concentration (mg/mL) was used to calculate EC_50_ value of sweetener-receptor interaction by nonlinear regression. EC_50_ is the measure of concentration and shows the amount of sweetener required to elicit the half-maximal responses; the lower the EC_50_, the more potent (sweet). Intensity (ΔF/F) at EC_50_ concentration refers to the maximal response to a sweetener. The higher ΔF/F, higher the intensity.

Concentration–response curves of ΔF/F to a range of concentrations of the compound were determined and used to calculate EC_50_ values of sweetener-receptor interaction. Mock-transfected cells did not show any calcium response when exposed to the same sweetener compound. Results show that SMF at low concentrations activates pig T1R2−T1R3. The potency of SMF activation is shown by EC_50_ = 0.50 mg/mL (500 ppm) (Figure 1).

### 3.2. SMF Included in the Feed of Weaning Piglets Upregulates GLUT2 and SGLT1 Expression with a Similar Magnitude

**Figure 2:**
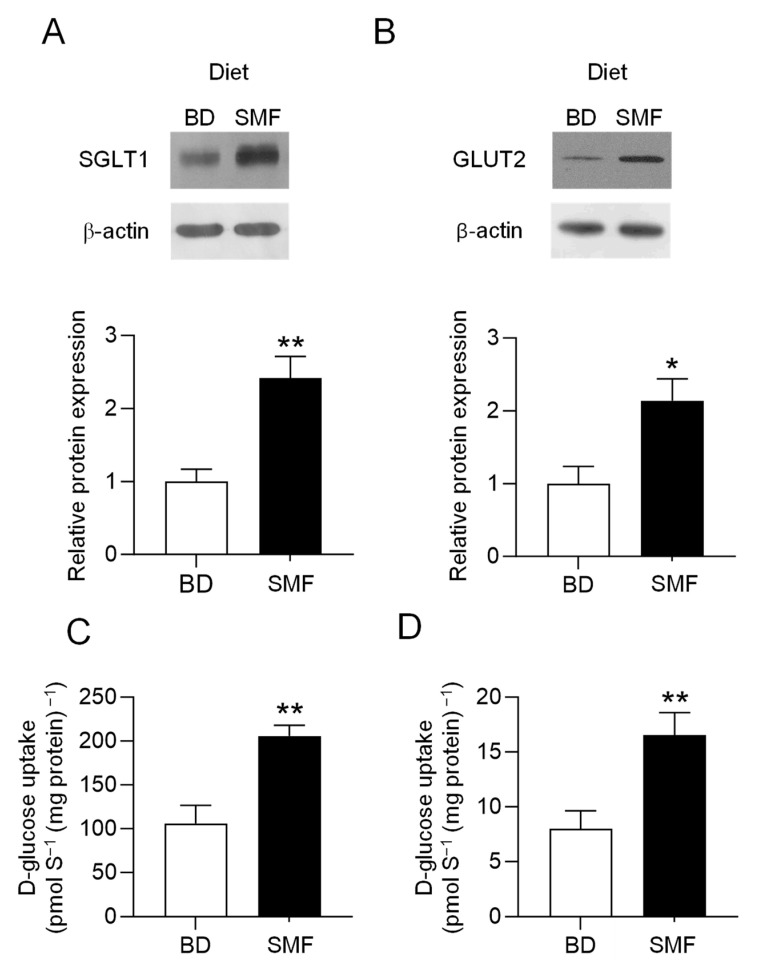
Increased SGLT1 and GLUT2 protein abundance and glucose transport in response to inclusion of SMF in piglets’ diet. Piglets were maintained on either a basal diet or the basal diet supplemented with SMF. SGLT1 and GLUT2 protein abundance was measured by western blotting and the SGLT1 or GLUT2 mediated glucose transport into BBMV or BLMV, respectively, were assessed by rapid filtration technique. (**A**) upper panel shows western blots for SGLT1 protein abundance in BBMV isolated from the proximal small intestine of piglets fed either the basal diet or the basal diet supplemented with SMF. Lower panel depicts densitometric scan of western blots of SGLT1 protein abundance normalised to β-actin protein, in piglets fed the basal diet (□) or basal diet + SMF (■). (**B**) upper panel shows western blots for GLUT2 protein abundance in BLMV isolated from the proximal small intestine of the same piglets fed either the basal diet or the basal diet supplemented with SMF. Lower panel shows densitometric scan of western blots of GLUT2 protein abundance normalised to that of β-actin, in piglets fed the basal diet (□) or basal diet + SMF (■). (**C**). Initial rates of Na^+^-dependent [U^14^C]-D-glucose uptake into BBMV isolated from the proximal small intestine of piglets maintained either on the basal diet (□) or basal diet + SMF (■). (**D**) Initial rates of [U^14^C]-D-glucose uptake into BLMV isolated from the same proximal small intestine of piglets on basal diet (□) or basal diet with SMF (■). Data were generated in triplicate, with *n* = 6–8 animals in each group. Statistical significance was determined by Student’s unpaired two-tailed *t*-test indicated by * *p* < 0.05; ** *p* < 0.01. Full-size western blots are shown in Supplementary Figure S2.

**Figure 3:**
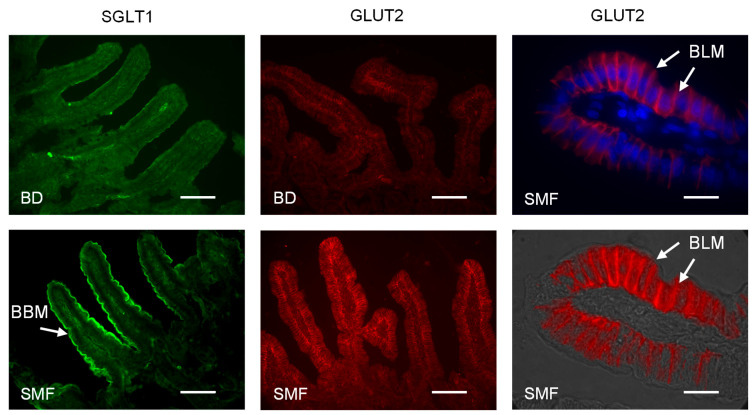
Immunofluorescent localisation of SGLT1 and GLUT2. Sections of the proximal small intestine of piglets maintained on either the basal diet (BD) or the basal diet supplemented with SMF (BD + SMF) were stained using antibodies to SGLT1 (green) and GLUT2 (red). Merged images at higher magnification show GLUT2 and the nuclear marker, DAPI (**right upper panel**), and a bright field image (**right lower panel**) demonstrate basolateral location of GLUT2 protein. Images are at 200× magnification (SGLT1, GLUT2) and 1000× magnification (GLUT2). BBM = brush border membrane; BLM = basolateral membrane. Scale bar = 20 μm for 200× images and 100 μm for 1000× images.

When piglets were maintained on a basal diet containing SMF, there was a 2-fold (*p* = 0.0019) increase in Na^+^-dependent glucose transport and a 2.2-fold (*p* = 0.0012) increase in SGLT1 protein abundance measured in BBMV isolated from the proximal small intestine. There was also a coordinated 2.1-fold (*p* = 0.04) increase in GLUT2 protein abundance and glucose transport (*p* = 0.0085) determined using BLMV isolated from proximal intestinal tissues of the same piglets fed the basal diet containing SMF (Figure 2). Full-size western blot images are shown in Figure S2. By immunohistochemistry, we show that SGLT1 and GLUT2 proteins are expressed on the luminal and basolateral membranes of absorptive enterocytes along the crypt-villus axis, respectively (Figure 3). The intensity of labelling (abundance) for SGLT1 and GLUT2 proteins is enhanced in response to the inclusion of SMF in the feed of weaning piglets (Figure 3).

### 3.3. Inclusion of SMF in the Feed of Weaning Piglets Enhances Animal Performance

Absorption of glucose via SGLT1 stimulates electrolyte (NaCl) and water absorption. In humans, this finding has been used in oral rehydration therapy, the safest and most effective remedy for treating life-threatening diarrhoea induced by agents such as *Vibrio cholerae* and *Escherichia coli* [33,34]. In weaning animals, diarrhoea, and consequent malnutrition and dehydration are major causes of mortality [35,36]. Inclusion of the plant-based sweetener formulation, SMF provides increased capacity for glucose, NaCl and water absorption preventing intestinal disorders [36]. With current trends aimed at decreasing the use of antibiotics, feed additives, such as SMF, that can improve the health and performance of weaning animals is highly desirable. We have shown here that when SMF was included in the diet, piglets’ weight increased by 19.6% and feed utilisation was improved. There was also a significant reduction (−3.3%) in the feed conversion ratio compared to controls (Table S3). Feed conversion ratio is the ratio of consumed feed over an animal’s weight gain [37]. In a large-scale feeding trial using 80 piglets, it was also shown that the inclusion of SMF in the diet led to a significant increase in final body weight and average daily gain over the course of the trial (*p* < 0.05) (Table S4), enhancing animal performance and health.

### 3.4. Addition of SMF in the Feed of Weaning Piglets Results in Increased Villus Height

**Figure 4:**
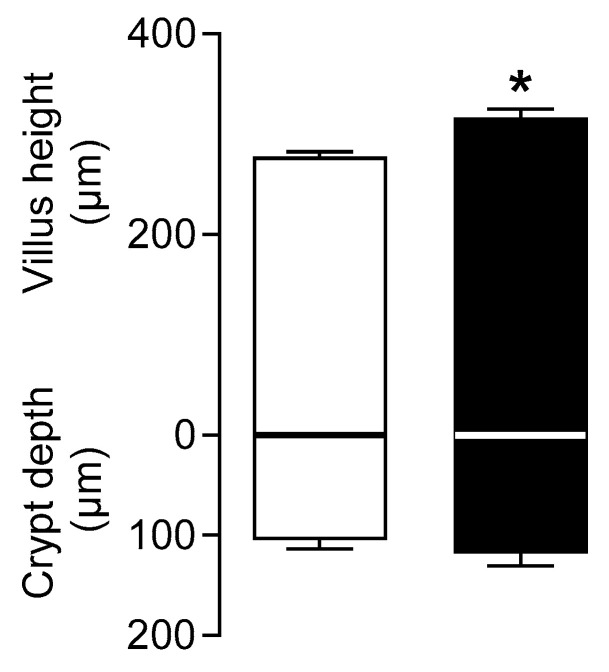
Morphometric analysis of the piglets’ intestine. Villus height and crypt depths in pig proximal small intestine fed with the basal diet (□) or basal diet supplemented with SMF (■) shown as histograms, in µm ± SEM; *n* = 4 animals. Statistically significant results were determined using Student’s two-tailed *t*-test where * = *p* < 0.05.

Morphometric analysis indicated that there was a 1.14-fold (*p* < 0.05) increase in villus height in the proximal small intestine when piglets were fed the diet supplemented with SMF compared to piglets fed the basal diet alone (Figure 4).

### 3.5. Direct Involvement of T1R2–T1R3 in Sweetener-Induced Upregulation of GLUT2 Expression

We have previously shown that artificial sweetener, sucralose activates pig T1R2–T1R3, but cyclamate and aspartame do not. Amino acid substitutions in binding sites for cyclamate and aspartame on pig T1R3 and T1R2 sequences, respectively, are responsible for the lack of T1R2–T1R3 activation [26]. We have previously demonstrated that cyclamate and aspartame do not upregulate SGLT1 activity and expression when included in piglets’ drinking water [26].

**Figure 5:**
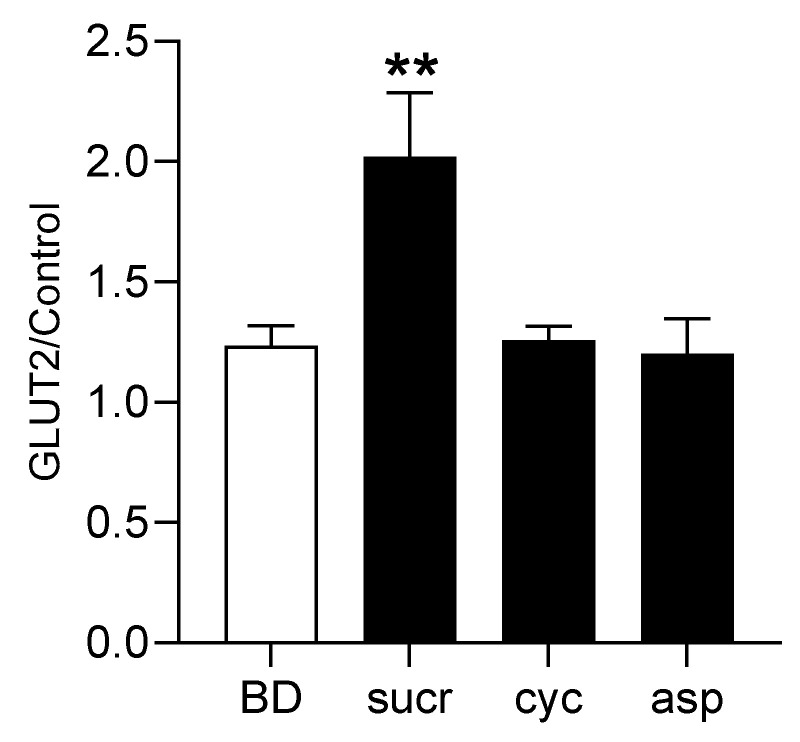
Expression of GLUT2 mRNA in the mid-small intestine of pigs fed a basal diet (**BD**) and sweeteners 2 mM sucralose (**sucr**), 10 mM cyclamate (**cyc**) or 1 mM aspartame (**asp**) included in their drinking water. The steady-state level of GLUT2 mRNA was normalised to the average expression of β-actin (ACTB), B2M and POLR2A mRNA (used as loading controls). Statistical analysis was carried out using a One-way ANOVA with a Dunnett’s multiple comparisons test where ** = *p* < 0.01.

To determine further, the involvement of sweet receptors in regulating GLUT2 expression, we maintained piglets on the basal diet or basal diet with their drinking water supplemented with 2 mM sucralose, 1 mM cyclamate or 1 mM aspartame. The expression of GLUT2 mRNA was assessed by quantitative PCR. There was a 1.7-fold (*p* = 0.0068) increase in GLUT2 mRNA expression in the proximal small intestine of piglets when sucralose was included in the drinking water compared to piglets consuming water alone. Cyclamate and aspartame had no effect on GLUT2 expression (Figure 5), demonstrating that sweeteners that do not activate pig T1R2–T1R3 have no effect on GLUT2 upregulation.

There were no statistical differences in the amount of water consumed by the piglets given either plain water or water containing sweeteners during the trial (Table S5).

### 3.6. Expression of GLUT2 in the Intestine of T1R3, G_α_-Gustducin Knockout Mice Is Not Responsive to Sweetener Intake Compared to Wild-Type Mice

**Figure 6:**
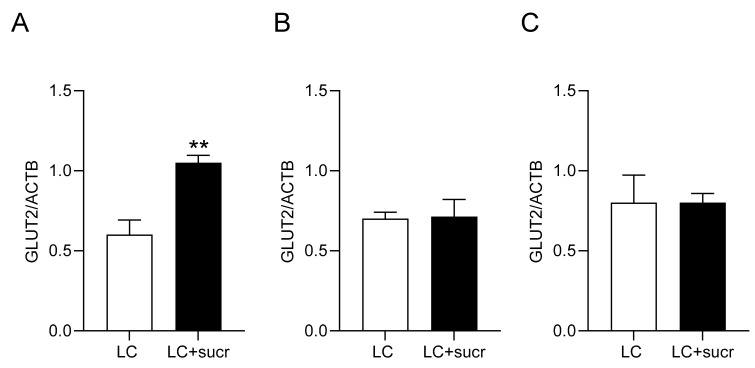
Steady-state levels of GLUT2 mRNA in the small intestine of either wild-type mice (**A**) or T1R3- (**B**) or α-gustducin (**C**)-knockout mice. Mice were fed a low-carbohydrate (1.9% sucrose, **LC**, □) diet or an LC diet and had their drinking water supplemented with sucralose (**LC + sucr, 2 mM**, ■). The abundance of GLUT2 mRNA was normalised to that of β-actin (ACTB) mRNA (used as loading control). *n* = 3 animals per group, statistical analysis was carried out using a student’s unpaired *t*-test where ** = *p* < 0.01.

In wild-type mice consuming 2 mM sucralose-sweetened water, there was a 1.8-fold increase (*p* = 0.0045) in GLUT2 mRNA as compared with wild-type controls (Figure 6). In contrast, in response to supplementation with sucralose, neither the G_α_-gustducin nor the T1R3 knockouts showed an enhancement in GLUT2 mRNA abundance (Figure 6). The data provide further evidence for the involvement of T1R2–T1R3 in GLUT2 upregulation.

### 3.7. Glucagon-like Peptide-2 Participates in the Pathway Regulating GLUT2 Expression

**Figure 7:**
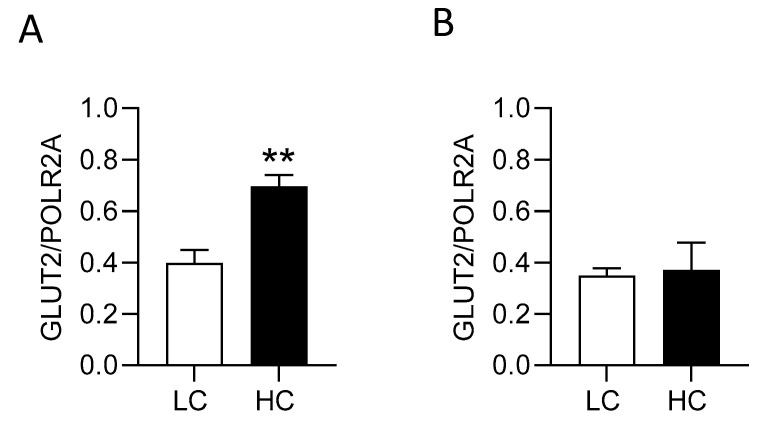
Steady-state levels of GLUT2 mRNA in the small intestine of wild-type mice (**A**) and GLP-2R knockout mice (**B**). Mice were fed either a low-carbohydrate (1.9% sucrose, **LC**, □) or a high-carbohydrate (69.8% sucrose, **HC**, ■) diet. The abundance of GLUT2 mRNA was normalised to that of RNA polymerase II (POLR2A) (used as loading control). *n* = 6–8 animals per group, statistical analysis was carried out using a Student’s unpaired *t*-test where ** = *p* < 0.01.

We have shown previously that GLP-2 is a component of the pathway regulating monosaccharide-induced upregulation of SGLT1 [10]. For GLP-2 to exert its effect, it must bind to its receptor GLP-2R. The receptor is expressed in enteric neurons [10,38,39]. Here we show that in wild-type mice fed a high sucrose diet, there was a 1.8-fold (*p* = 0.0015) increase in GLUT2 mRNA abundance in the proximal small intestine compared to wild-type mice fed a low-sucrose-containing diet. GLP-2R knockout mice fed either the low or high-carbohydrate diets had similar levels of GLUT2 mRNA observed in wild-type mice fed the low-sucrose diet (Figure 7), suggesting that GLP-2 participates in the pathway regulating carbohydrate-induced regulation of GLUT2 expression.

### 3.8. Electric Field Stimulation of Mouse Small Intestine Enhanced GLUT2 Expression

**Figure 8:**
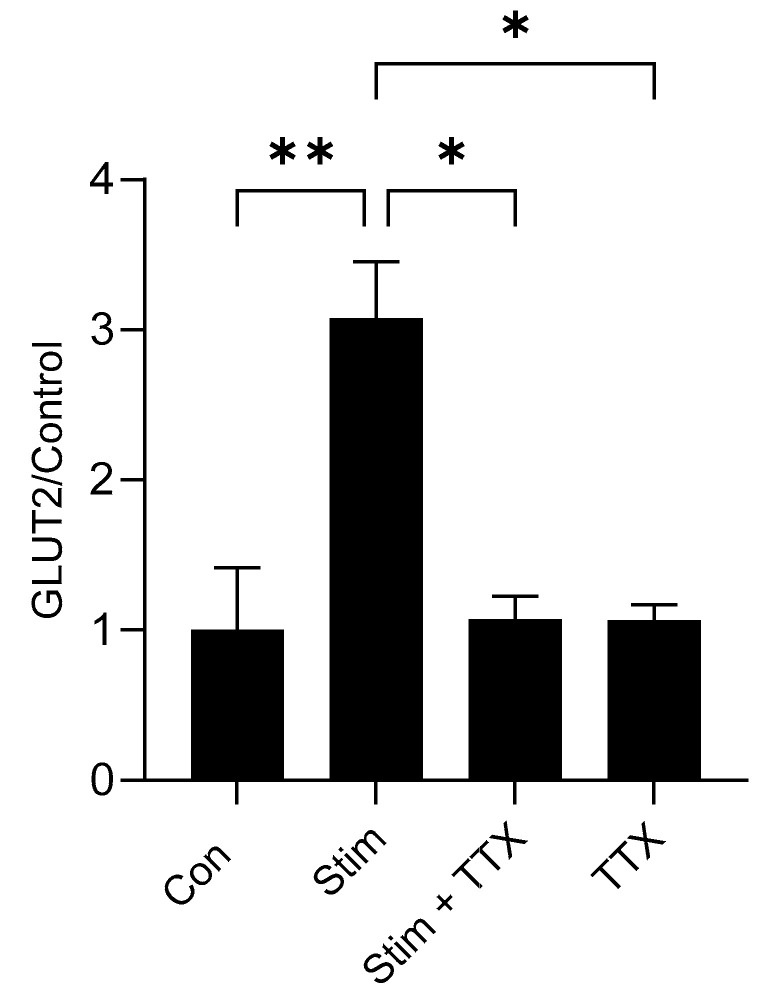
Electric field stimulation enhances GLUT2 mRNA expression. Mouse intestinal tissue loops were stimulated continuously for 20 min, using frequency of 20 Hz, with 0.3 ms duration, 1 ms delay and 50 V in the absence/presence of TTX or remained unstimulated as described in Methods. Steady-state levels of GLUT2 mRNA as assessed by qPCR, in unstimulated tissue (Con), stimulated tissue (Stim), tissue stimulated for 20 min in the presence of the 10 µM tetrodotoxin (Stim + TTX) or unstimulated tissue in the presence of 10 µM tetrodotoxin (TTX) (*n* = 3–7). The abundance of GLUT2 mRNA was normalised to the average expression of β-actin (ACTB), B2M and POLR2A mRNA (used as loading controls). Data are expressed as means ± SEM. *p* values were determined using a One-way ANOVA with a Dunnett’s multiple comparisons test where * *p* < 0.05; ** *p* < 0.01.

In response to 20 min of electric field stimulation (EFS), there was a 3.1-fold (*p* = 0.0054) increase in GLUT2 mRNA levels compared to unstimulated tissue (Figure 8). This increase in GLUT2 expression was eliminated when the tissue was preincubated with the neuronal sodium channel blocker TTX (Figure 8). TTX alone had no effect on GLUT2 expression in unstimulated tissue.

## 4. Discussion

Transcellular transport of glucose in the intestine, via apically located SGLT1 and basally residing GLUT2, is important for the provision of energy and maintenance of glucose homeostasis.

It has been shown that in the intestine of patients with type 2 diabetes, there is an enhanced expression and activity of SGLT1 and GLUT2, and that this increased expression of SGLT1 and GLUT2 is independent of changes in blood glucose or insulin levels, and likely due to alterations in mechanisms and signalling pathways involved in the regulation of these sugar transporters [30]. It has also been reported that in subjects with type 2 diabetes, there is a decrease in the expression of T1R2 and downstream signalling elements, suggesting deregulation in the pathway underlying intestinal glucose sensing and signalling [40]. A better understanding of mechanisms involved in glucose sensing and signalling involved in the regulation of intestinal glucose transport will further our understanding of the deregulation of this process in pathology. The knowledge can provide novel therapeutic targets for modulating the gut’s capacity to absorb sugars, with implications for the prevention of diet-related disorders including diabetes and obesity.

It was shown, for the first time, that the lingual epithelium sweet taste receptor T1R2–T1R3 is expressed in intestinal enteroendocrine cells [41]. Subsequently, using T1R3 and α-gustducin knockout mice, it was demonstrated that the gut-expressed sweet taste receptor is required for the regulation of SGLT1 expression, and that the receptor is activated by glucose and artificial sweeteners [7]. Recent experimental evidence has indicated that sensing of sweet compounds by T1R2–T1R3 expressed in L-enteroendocrine cells triggers a downstream signalling pathway resulting in GLP-2 secretion, and this gut hormone via a neuro-paracrine pathway enhances the half-life of SGT1 mRNA leading to upregulation of SGLT1 in absorptive enterocytes [10]. However, little information is available on the pathway regulating the activity and expression of intestinal GLUT2.

Mace et al., 2007 have reported that sensing of glucose and artificial sweeteners increases intestinal glucose absorption by rapid insertion of GLUT2 to the apical membrane of enterocytes, and the underlying mechanism is through glucose/sweetener-induced increase in intracellular calcium concentration [42]. Smith et al., 2018 have proposed that T1R2–T1R3 mediated glucose sensing in the proximal intestine regulates the rate of glucose transport through regulation of GLUT2 trafficking from basolateral to the apical membrane of enterocytes, and that GLUT2 translocation and glucose transport are dependent on GLP-2 secretion and activation of enteric nervous system [43]. However, no further studies have been published to show how an increase in intracellular calcium concentration, or GLP-2 secretion can result in the insertion/translocation of GLUT2 to the apical membrane. Wright’s laboratory has shown that in glucose-galactose malabsorption, resulting from mutations within the SGLT1 gene [44], human infants cannot absorb glucose or galactose. The presence of GLUT2 on the luminal membrane would have forestalled the chronic and potentially fatal diarrhoea, refuting the existence of GLUT2 on the luminal membrane.

In this paper, using heterologous expression assay we show that SMF at low concentrations, EC_50_ = 0.50 mg/mL (500 ppm), activates pig T1R2–T1R3. Furthermore, we demonstrate that SMF included in the diet of weaning piglets, results in a 2-fold increase in GLUT2 protein abundance and glucose transport in basolateral membrane vesicles isolated from proximal intestinal tissues of piglets fed the basal diet containing SMF. There is also a coordinated 2-fold increase in Na^+^-dependent glucose transport and SGLT1 protein abundance in the brush border membrane.

By immunohistochemistry we find that SGLT1 and GLUT2 proteins are exclusively expressed on the luminal and basolateral membrane of absorptive enterocytes along the crypt-villus axis, respectively, irrespective of dietary composition. The intensity of labelling for SGLT1 and GLUT2 proteins is enhanced in response to the inclusion of SMF in the feed of weaning piglets. To examine direct involvement of T1R2–T1R3 in sweetener-induced upregulation of GLUT2 expression, we show that (i) the sweet compounds that do not activate pig T1R2–T1R3 [26] had no effect on GLUT2 upregulation, and (ii) there was no increase in GLUT2 expression in G_α_-gustducin and T1R3 knockout mice in response to feed supplemented with sucralose (in comparison to wild-type mice).

GLP-2 is an intestinal trophic endocrine hormone known to increase intestinal growth reflected in enhancement in villus height [45,46]. In this study, morphometric analysis shows that there is a significant increase in villus height in the intestine of piglets fed a diet including SMF. This is good evidence for GLP-2 release in response to the activation of gut-expressed T1R2–T1R3 by SMF. For GLP-2 to exert its effect, it must bind to its receptor. It is widely recognised that the GLP-2 receptor is expressed in enteric neurons [38,39]. The binding of GLP-2 to its receptor transmits a neuronal spike to the epithelium. This response can be blocked by the voltage-gated sodium channel inhibitor tetrodotoxin (TTX) [38]. We show here that in response to electric stimulation of the mouse intestine, there is a 3.1-fold (*p* = 0.0054) increase in GLUT2 mRNA expression compared to unstimulated tissue. Pre-treatment of mouse intestinal tissue with TTX abolishes upregulation of GLUT2 mRNA expression in response to electric field stimulation indicating that the enteric nervous system participates in the pathway regulating GLUT2 expression. We further show that, unlike wild-type mice, in GLP-2R knockout mice there was no upregulation of GLUT2 mRNA expression in response to a high-carbohydrate diet. The lack of response in GLP-2 receptor knockout mice is not attributed to the altered profile of GLP-2 expression along the crypt-villus axis. It is due to the absence of the receptor for GLP-2 required for this gut hormone to bind to exert its biological effect. The data suggest that GLP-2 is a constituent of the pathway regulating carbohydrate-induced regulation of GLUT2 expression.

Whilst GLP-2 is known to enhance intestinal tissue growth, its action on increasing intestinal nutrient transport appears to be beyond the control of gut growth [10,18]. In support of this proposition, we show here that while there is a 1.14-fold increase in villus height in piglets maintained on SMF, there is a 2-fold increase in SGLT1 and GLUT2 expression, suggesting that GLP-2 action on increasing intestinal glucose transport is beyond the control of gut growth.

The understanding of the regulatory pathway controlling the transcellular transport of intestinal glucose transport allows the identification of targets for controlling the capacity of the gut to absorb glucose. This has an attendant promise for preventing and/or treating conditions such as weaning-related intestinal disorders, diabetes and obesity.

## 5. Conclusions

We aimed to determine if the gut-expressed sweet taste receptor, T1R2−T1R3, and the carbohydrate-responsive gut hormone, GLP-2 and its receptor, are the components of the pathway regulating GLUT2 expression. We used a wide range of experiments assessing the participation of T1R2−T1R3 in the regulatory pathway. A plant-based sweetener formulation, SMF, that activates T1R2−T1R3, when included in piglets’ diet led to a significant increase in GLUT2 expression compared to controls. The sweeteners that do not activate pig T1R2−T1R3, failed to upregulate GLUT-2 expression. In knockout mice, in which the genes for the sweet receptor subunit T1R3 and the associated G-protein gustducin were deleted, there was no upregulation of GLUT2 expression in response to sucralose supplementation. In contrast, sucralose, which activates T1R2−T1R3, when included in the drinking water of wild-type mice led to an increase in GLUT2 expression. To examine the involvement of the gut hormone GLP-2, we demonstrated that in contrast to the wild-type mice, in GLP-2 receptor knockout mice there was no upregulation in GLUT2 expression when maintained on a high-carbohydrate diet. We conclude that luminal sweet sensing via T1R2–T1R3 and the gut hormone GLP-2 and enteric nervous system are components of the pathway regulating GLUT2 expression.

## Data Availability

The data supporting the findings of this study are available from the corresponding author S.P.S.-B. upon request or online through https://www.liverpool.ac.uk/people/soraya-shirazi-beechey Publications. This is accessible from 1 May 2025.

## Supplementary Materials

The following supporting information can be downloaded at: https://www.mdpi.com/article/10.3390/nu17091547/s1, Figure S1: Immunocytochemical detection of transfected porcine sweet taste receptor subunits; Figure S2: Western blot of SGLT1 protein in BBMV and GLUT2 protein in BLMV isolated from piglet proximal small intestine; Table S1: Composition and analysis of piglets’ diets; Table S2: Composition of diets, D1, D2, D3, and D1, D2 and D3 + SMF diets; Table S3. Weight gain, feed and water intake and feed conversion ratio for BD and BD + SMF fed piglets; Table S4. Weight, weight gain, feed intake and feed conversion ratio for control diet (CD) and CD + SMF fed piglets over 28 days; Table S5: Weight gain, feed and water intake for Basal Diet (BD) plus sweeteners (sucralose, cyclamate and aspartame) included in the piglets’ drinking water.

## Abbreviations

The following abbreviations are used in this manuscript:

ACTB	β-actin
BBMV	Brush border membrane vesicle
BD	Basal diet
BLMV	Basolateral membrane vesicle
B2M	β2-microglobulin
CD	Control diet
DAPI	4’,6-diamidino-2-phenylindole
DTT	Dithiothreitol
EDTA	Ethylenediaminetetraacetic acid
FITC	Fluorescein isothiocyanate
GLP-1	Glucagon-like peptide 1
GLP-2	Glucagon-like peptide 2
GLUT2	Glucose transporter 2
OCT	Optimal cutting temperature
PACAP	Pituitary adenylate cyclase-activating polypeptide
PMSF	Phenylmethylsulfonyl fluoride
POLR2A	RNA polymerase 2 subunit RPB1
PVDF	Polyvinylidene difluoride
SDS	Sodium dodecyl sulfate
SGLT1	Sodium/glucose co-transporter 1
TTX	Tetrodotoxin
T1R2	Taste 1 receptor 2
T1R3	Taste 1 receptor 3
VIP	Vasoactive intestinal peptide
