# Supplementary material for: Luminal Sweet Sensing and Enteric Nervous System Participate in Regulation of Intestinal Glucose Transporter, GLUT2
Source: Nutrients. 2025 Apr 30;17(9):1547. doi: 10.3390/nu17091547 (PMC12073725; doi:10.3390/nu17091547)
Supplement: Supplementary file 1 [file nutrients-17-01547-s001.zip › nutrients-3568729-supplementary.pdf]

Supplementary Material

1 Supplementary Tables

Table S1. Composition and analysis of piglet diets.

	Diet	
	**BD**	**BD + SMF**
**Composition (g/kg)**		
Porridge Oats	170.0	170.0
Micro Ground Wheat	372.5	372.5
Micro Ground Maize	100.0	100.0
Potato Protein	25.0	25.0
Fullfat Soy Bean Extruded	200.0	200.0
Provimi White Fish	75.0	75.0
L-Lysine HCl	5.5	5.5
DL-Methionine	2.5	2.5
L-Threonine	2.0	2.0
Soya Oil	24.0	24.0
Limestone Trucal 52	3.0	3.0
Monocalcium Phosphate	10.0	10.0
Salt	5.0	5.0
Weaner Trials Supplement	5.0	5.0
Piglet Flavour (Vanilla)	0.5	0.5
SMF	—	0.10
**Analysis (%)**		
Crude Protein	22.1	22.1
Oil	8.4	8.4
Fibre	2.9	2.9
Ash	5.4	5.4
Starch	38.9	38.9
Sugar	2.8	2.8
Lactose	—	—
Salt	0.6	0.6
Essential Fatty Acids	4.7	4.7
Lysine	1.6	1.6
Methionine	0.6	0.6
Threonine	1.0	1.0
Calcium	1.0	1.0
Phosphate	0.8	0.8
**Digestible Energy (kJ/g)**	**16.76**	**16.76**

Composition and analysis of BD=Basal diet and BD + SMF = the same diet but supplemented with the sweetener formulation, SMF at a concentration of 0.10 g per kg of feed. Both diets are isoenergetic.

Table S2. Composition of diets, D1, D2, D3 and D1, D2 and D3 + SMF

	Phase 1		Phase 2		Phase 3	
	D1	D1+ SMF	D2	D2 + SMF	D3	D3 + SMF
**Composition (%)**						
Corn ground	18.95	18.95	32.05	32.05	55.24	55.24
Whey permeate	15.73	15.73	12.50	12.50	-	-
Whey protein conc	4.78	4.78	-	-	-	-
47.5% soybean meal	29.23	29.23	25.95	25.95	22.40	22.40
Oat, flour/meal feed	15.00	15.00	-	-	-	-
Oat groat, steam	-	-	7.00	7.00	-	-
Grease mx choice white	4.50	4.50	3.50	3.50	-	-
Phosphate monocal 21	2.25	2.25	1.20	1.20	1.20	1.20
Dist dr grn&sol	2.20	2.20	10.05	10.05	10.05	10.05
Calcium carbonate	0.74	0.74	1.07	1.07	0.99	0.99
Lysine-hcl, 98%	0.53	0.53	0.57	0.57	0.56	0.56
Salt	0.32	0.32	0.52	0.52	0.91	0.91
Mha 84% (methionine)	0.26	0.26	0.19	0.19	0.18	0.18
Threonine, l-98.5	0.25	0.25	0.13	0.13	0.20	0.20
Swine mix with trace Elements	0.28	0.28	0.29	0.29	0.27	0.27
Corn ground SMF premix equivalent	5.00	-	5.00	-	5.00	-
SMF premix*	-	5.00	-	5.00	-	5.00
**Nutrients**						
Protein %	20.58	20.58	19.28	19.28	18.04	18.04
Fat; crude %	7.03	7.03	6.50	6.50	6.05	6.05
Starch %	24.44	24.44	28.93	28.93	39.91	39.91
Adf %	3.27	3.27	4.50	4.50	4.86	4.86
Neut det. Fiber %	6.50	6.50	8.59	8.59	9.34	9.34
ME swine kcal/lb	1587.9820	1587.9820	1570.2320	1570.2320	1559.5400	1559.5400
Calcium %	0.9023	0.9023	0.8086	0.8086	0.7229	0.7229
Phosphorus %	0.9020	0.9020	0.6758	0.6758	0.6039	0.6039
Avail. lysine swine %	1.4968	1.4968	1.3510	1.3510	1.2314	1.2314

*premix based on corn ground and delivering 100 ppm of SMF in feed

Table S3. Weight gain, feed and water intake and feed conversion ratio for BD and BD + SMF fed piglets.

	Diet	
	BD	BD + SMF
Starting weight (kg)	7.86 ± 0.59	8.3 ± 1.99
End weight (kg)	9.4 ± 0.84	10.14 ± 2.51
Average Daily Weight gain (g)	128.6 ± 49.82	144.6 ± 48.1
Average Daily feed intake (g)	472.9 ± 14 g	372.1 ± 58
Feed conversation ratio (FCR)	4.1 ± 1.4	2.7 ± 0.5
Daily water intake (mL per pen)	1860.4 ± 513.7	1827.1 ± 597.7

* Starting, end weights and average daily gain were measured per piglet. All values are stated ± SD.

** Daily water intake and feed intake were measured per pen (two piglets per pen).

Table S4. Weight, weight gain, feed intake and feed conversion ratio for control diet (CD) and CD + SMF fed piglets over 28 days.

	Dietary treatments	
	BD	BD + SMF
Starting weight (kg)	6.38 ± 1.05	6.42 ± 0.99
End weight (kg)	15.30 ± 1.72	16.24 ± 1.92*
Average Daily Weight gain (g)	318.6 ± 32	350.9 ± 41*
Average Daily feed intake (g)	488.1 ± 58	518.6 ± 63
Feed conversation ratio (FCR)	1.530 ± 0.063	1.480± 0.098

*P<0.05

Table S5. Weight gain, feed and water intake for BD plus sweeteners (sucralose, cyclamate and aspartame) included in the piglets' drinking water.

	Diet			
	BD	sucralose	cyclamate	aspartame
Starting weight (kg)*	7.53 ± 0.49	8.3 ± 0.49	7.06 ± 0.37	6.8 ± 0.40
End weight (kg)*	7.14 ± 0.52	8.23 ± 0.58	6.9 ± 0.62	7.0 ± 0.54
Daily feed intake** (g per pen)	336.5 ± 400.5	495.6 ± 410.9	387.1 ± 163.5	587.6 ± 411.2
Daily water intake** (mL per pen)	967.63 ± 434.8	1212.5 ± 423.1	1258.3 ± 390.6	1437.5 ± 474.5

* Starting and final weights were measured per piglet. All values are stated ± SD.

** Daily water intake and feed intake were measured per pen (two piglets per pen).

2 Supplementary Figures

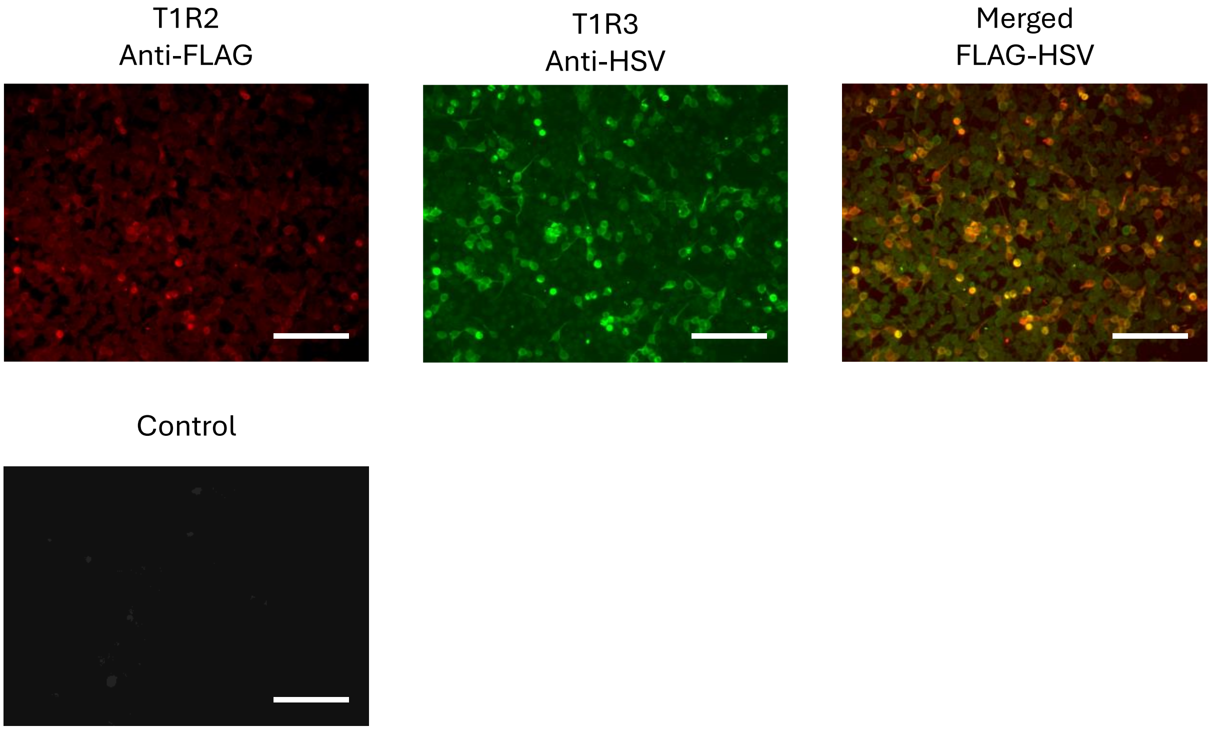

Supplementary Figure 1. Immunocytochemical detection of transfected porcine sweet taste receptor subunits. HEK293PEAKrapid $G_{\alpha 15}$ cells were transfected with cDNA coding for pig T1R2 and T1R3 subunits and probed with antibodies directed against the C-terminal tags FLAG and HSV. Cells express T1R2 (green), T1R3 (red) and when overlaid showing co-expression of T1R2 and T1R3 (orange) in the same cell and at the plasma membrane. Cells transfected with empty vector served as a negative control. Images are 100X magnified. Scale bar = 100 µm.

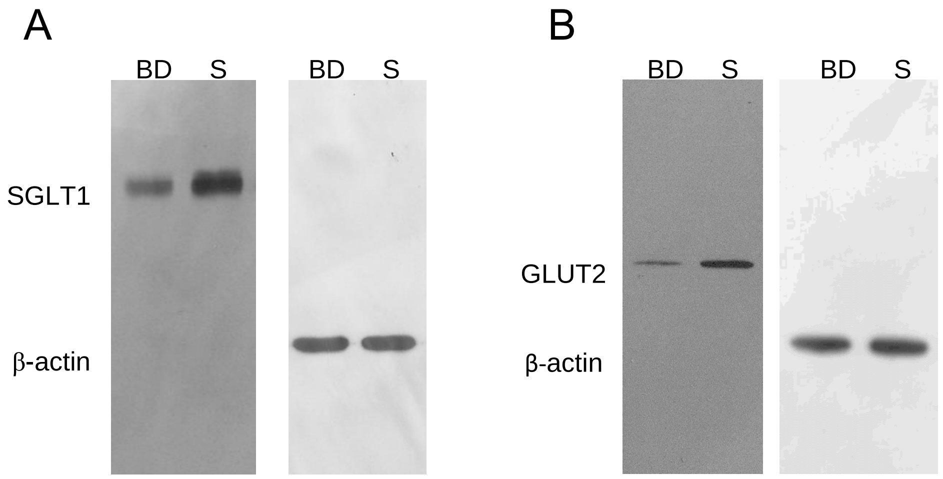

Supplementary Figure 2. Western blotting for SGLT1 in BBMV and GLUT2 in BLMV in the piglet proximal small intestine. BBMV and BLMV were isolated from the mid-small intestine of piglets fed either the basal diet (BD) or basal diet supplemented with SMF (S). BBMV (A) and BLMV (B) were separated on 8% (w/v) SDS-PAGE gels and transferred to PVDF membranes. Membranes were blotted for either SGLT1 (A) or GLUT2 (B). Images present are full size blots corresponding to those present in figure 2.
